# The role of π–π stacking and hydrogen-bonding inter­actions in the assembly of a series of isostructural group IIB coordination compounds

**DOI:** 10.1107/S2053229618018314

**Published:** 2019-01-25

**Authors:** Taraneh Hajiashrafi, Roghayeh Zekriazadeh, Keith J. Flanagan, Farnoush Kia, Antonio Bauzá, Antonio Frontera, Mathias O. Senge

**Affiliations:** a Department of Chemistry, Faculty of Physics and Chemistry, Alzahra University, PO Box 1993891176, Tehran, Iran; b School of Chemistry, SFI Tetrapyrrole Laboratory, Trinity Biomedical Science Institute, Trinity College Dublin, The University of Dublin, 152–160 Pearse Street, Dublin 2, Ireland; c Department of Chemistry, University of the Balearic Islands, Carretera de Valldemossa km 7.5, 07122 Palma de Mallorca, Illes Balears, Spain

**Keywords:** π-stacking, hydrogen bonding, noncolvalent inter­actions, metallo­supra­molecular assembly, crystal structure, coordination compound, group IIB

## Abstract

The supra­molecular chemistry of coordination compounds has become an im­portant research domain of modern inorganic chemistry. Herein, six iso­structural group IIB coordination compounds containing a 2-{[(2-meth­oxy­phen­yl)imino]­meth­yl}phenol ligand, namely di­chlorido­bis­(2-{(*E*)-[(2-meth­oxy­phen­yl)aza­niumyl­idene]meth­yl}pheno­lato-κ*O*)zinc(II), [ZnCl_2_(C_28_H_26_N_2_O_4_)], **1**, di­iodido­bis­(2-{(*E*)-[(2-meth­oxy­phen­yl)aza­niumyl­idene]meth­yl}pheno­lato-κ*O*)zinc(II), [ZnI_2_(C_28_H_26_N_2_O_4_)], **2**, di­bromido­bis­(2-{(*E*)-[(2-meth­oxy­phen­yl)aza­n­iumyl­idene]meth­yl}pheno­lato-κ*O*)cadmium(II), [CdBr_2_(C_28_H_26_N_2_O_4_)], **3**, di­iodido­bis­(2-{(*E*)-[(2-meth­oxy­phen­yl)aza­niumyl­idene]meth­yl}pheno­lato-κ*O*)cadmium(II), [CdI_2_(C_28_H_26_N_2_O_4_)], **4**, di­chlorido­bis­(2-{(*E*)-[(2-meth­oxy­phen­yl)aza­niumyl­idene]meth­yl}pheno­lato-κ*O*)mercury(II), [HgCl_2_(C_28_H_26_N_2_O_4_)], **5**, and di­iodido­bis­(2-{(*E*)-[(2-meth­oxy­phen­yl)aza­niumyl­idene]meth­yl}pheno­lato-κ*O*)mercury(II), [HgI_2_(C_28_H_26_N_2_O_4_)], **6**, were synthesized and characterized by X-ray crystallography and spectroscopic techniques. All six compounds exhibit an infinite one-dimensional ladder in the solid state governed by the formation of hydrogen-bonding and π–π stacking inter­actions. The crystal structures of these compounds were studied using geometrical and Hirshfeld surface analyses. They have also been studied using M06-2X/def2-TZVP calculations and Bader’s theory of ‘atoms in mol­ecules’. The energies associated with the inter­actions, including the contribution of the different forces, have been evaluated. In general, the π–π stacking inter­actions are stronger than those reported for conventional π–π complexes, which is attributed to the influence of the metal coordination, which is stronger for Zn than either Cd or Hg. The results reported herein might be useful for understanding the solid-state architecture of metal-containing materials that contain *M* ^II^ *X*_2_ subunits and aromatic organic ligands.

## Abstract 2

This article describes the syntheses, crystal structures and theoretical calculations of six group IIB coordination compounds containing a 2-{[(2-meth­oxy­phen­yl)imino]­meth­yl}phenol ligand to provide further insight into the role of π-stacking and hydrogen bonding in metallo­supra­molecular assembly.

## Introduction   

Over the last two decades, the supra­molecular chemistry of metal-containing compounds has attracted intense attention, due not only to their fascinating structures (Holliday & Mirkin, 2001 ▸; Brammer, 2004 ▸), but also their potential applications in diverse fields such as medicine (McKinlay *et al.*, 2010 ▸; Reedijk, 2009 ▸), ion and mol­ecular recognition (Custelcean *et al.*, 2012 ▸; Busschaert *et al.*, 2015 ▸) and catalysis (Wang *et al.*, 2013 ▸; Wiester *et al.*, 2011 ▸).

The ultimate goal of supra­molecular chemistry is to understand the inherent complexities of the association mechanisms of mol­ecular and ionic building blocks organized through noncovalent inter­molecular inter­actions with pre­scribed properties and functions (Lehn, 1995 ▸; Steed & Atwood, 2013 ▸). In the context of metallo­supra­molecular chemistry (Braga & Grepioni, 2000 ▸; Braga *et al.*, 1998 ▸), hydrogen bonding (Reedijk, 2013 ▸; Azhdari Tehrani *et al.*, 2016 ▸) and halogen bonding (Khavasi *et al.*, 2015 ▸; Khavasi & Azhdari Tehrani, 2013 ▸; Li *et al.*, 2016 ▸) have been widely used so far to drive the self-assembly of coordination compounds, because of their directionality and versatility (Politzer *et al.*, 2010 ▸; Desiraju, 1998 ▸). However, there are some reports that provide evidence suggesting the crucial role of nondirectional inter­molecular inter­actions, such as π–π stacking (Khavasi & Azizpoor Fard, 2010 ▸; Janiak, 2000 ▸; Khavasi & Sadegh, 2014 ▸; Semeniuc *et al.*, 2010 ▸), for designing the supra­molecular architecture of metal-containing species in the solid state. In this regard, supra­molecular chemists and crystal engineers have explored and studied the use of noncovalent inter­actions as a key tool for constructing supra­molecular architectures of metal-containing building units in the solid state in which X-ray crystallography could provide a detailed picture of the supra­molecular structure (Desiraju, 2014 ▸; Blake *et al.*, 1999 ▸; Đaković *et al.*, 2018 ▸). These studies reveal an undeniable contribution of such noncovalent inter­actions to the organization and stabilization of the ultimate crystal structures. These studies also revealed that the ultimate supra­molecular architecture of self-assembled metal-containing compounds could be affected by various factors, such as ligand and metal geometries (Khavasi *et al.*, 2012 ▸; Hajiashrafi *et al.*, 2013 ▸), counter-ions (Schottel *et al.*, 2006 ▸; Zeng *et al.*, 2010 ▸) and reaction conditions (Khavasi & Mohammad Sadegh, 2010 ▸; Mahata *et al.*, 2009 ▸).

In continuation of our research aimed at understanding the role of noncovalent inter­actions in the fabrication and self-assembly of metal-containing building blocks (Hajiashrafi *et al.*, 2013 ▸, 2016 ▸; Kielmann & Senge, 2018 ▸), a series of coordination compounds, namely [Zn*L*_2_Cl_2_] (**1**), [Zn*L*_2_I_2_] (**2**), [Cd*L*_2_Br_2_] (**3**), [Cd*L*_2_I_2_] (**4**), [Hg*L*_2_Cl_2_] (**5**) and [Hg*L*_2_I_2_] (**6**), where *L* is 2-{[(2-meth­oxy­phen­yl)azaniumylidene]­meth­yl}phe­nolate, have been synthesized and characterized using X-ray crystallography and different spectroscopic techniques (see Scheme 1). Geometrical, Hirshfeld surface analysis and theoretical calculations reveal the importance of π–π stacking inter­actions, as well as hydrogen bonding, in governing the crystal packing of this series of isostructural metal-containing compounds.

## Experimental   

### Materials and apparatus   

Chemicals and reagents were purchased from commercial sources. 2-Hy­droxy­benzaldehyde, 2-meth­oxy­aniline and an­hydrous *M* ^II^ halides, where *M* is Zn, Cd and Hg, were pur­chased from Sigma–Aldrich and Merck, and used as received. The Schiff base ligand 2-{[(2-meth­oxy­phen­yl)imino]­meth­yl}phenol (*L*) was prepared according to a previously reported method (Song *et al.*, 2013 ▸). The IR spectra were recorded on a Nicolet FT–IR 100 spectrometer in the range 500–4000 cm^−1^ using the KBr disk technique. Elemental analyses (C, H and N) were performed using an ECS 4010 CHN-O made in Costech, Italy. Melting points were measured by an Electrothermal 9100 melting-point apparatus and corrected. The measurements were carried out using 10 mg of a powdered sample sealed in aluminium pans with a mechanical crimp.

### Computational methods   

The geometries of the complexes included in this study were computed at the M06-2X/def2-TZVP level of theory using the crystallographic coordinates within *TURBOMOLE 7.0* (Ahlrichs *et al.*, 1989 ▸). We have used the crystallographic coordinates instead of the optimized complexes because we are inter­ested in estimating the binding energies of several assemblies as they stand in the crystal structure, instead of investigating the most favourable geometry for a given complex. The inter­action energies were calculated with correction for the basis set superposition error (BSSE) by using the Boys–Bernardi counterpoise technique (Boys & Bernardi, 1970 ▸). The ‘atoms-in-mol­ecules’ (AIM) analysis of the electron density was performed at the same level of theory using the *AIMAll* program (Keith, 2013 ▸).
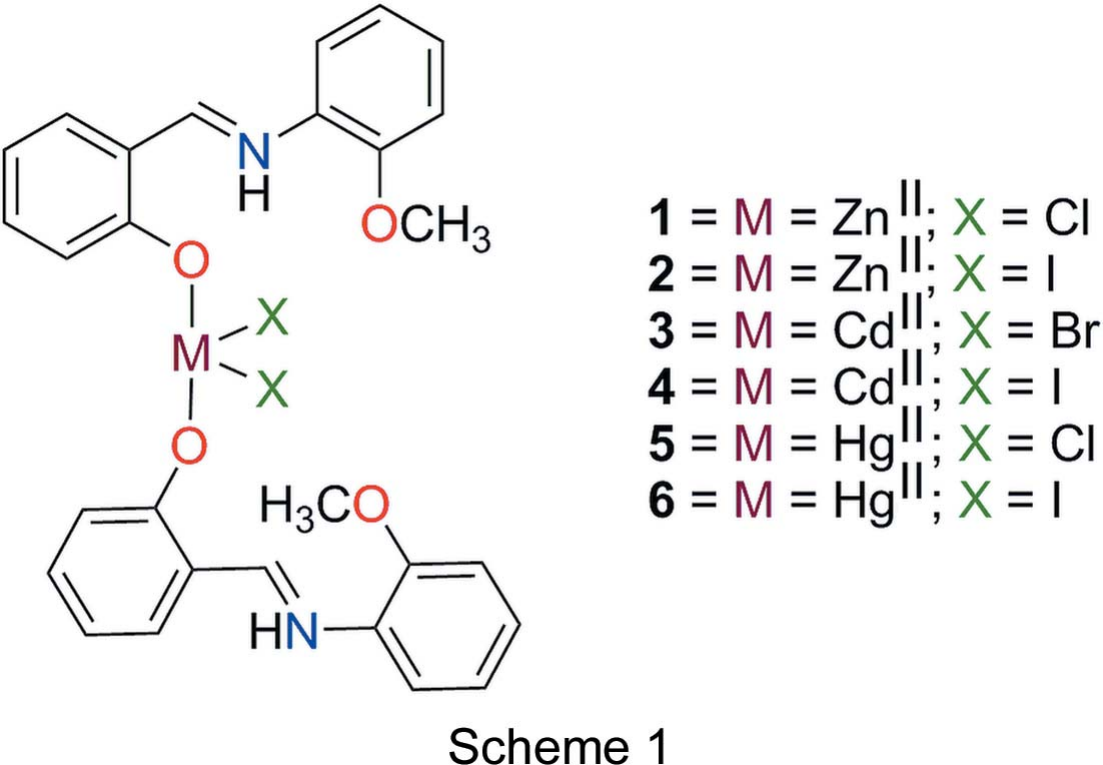


### Synthesis and crystallization   

The ligand 2-{[(2-meth­oxy­phen­yl)imino]­meth­yl}phenol (*L*) was utilized previously for the preparation of a number of coordination compounds (Song *et al.*, 2013 ▸; Gong *et al.*, 2014 ▸; Reddy *et al.*, 2003*a* ▸,*b* ▸; Li & Yuan, 2012 ▸). *L* was syn­thesized by reacting 2-hy­droxy­benzaldehyde (0.53 ml, 5 mmol) with 2-meth­oxy­aniline (0.56 ml, 5 mmol) in ethanol. After stirring for 30 min at 323 K, the ligand precipitated from the reaction mixture as an orange powder which was filtered off, washed several times with cold ethanol and normal hexane, and then dried under vacuum.

The six coordination compounds [Zn*L*_2_Cl_2_] (**1**), [Zn*L*_2_I_2_] (**2**), [Cd*L*_2_Br_2_] (**3**), [Cd*L*_2_I_2_] (**4**), [Hg*L*_2_Cl_2_] (**5**) and [Hg*L*_2_I_2_] (**6**) were synthesized by combining a solution of *MX*_2_ (0.1 mmol; *M* = Zn, Cd or Hg and *X* = Cl, Br or I) in methanol (5 ml) and a solution of *L* (0.2 mmol) in methanol (5 ml) with stirring. Each mixture was heated at 333 K for about 30 min. Reduction of the solvent volume resulted in the formation of a yellow-to-orange precipitate. The precipitate was filtered off, washed with methanol (3 × 2 ml) and then dried *in vacuo*. The solid was subsequently dissolved in boiling methanol, ethanol or aceto­nitrile (10 ml) and filtered. Upon slow evaporation of the filtrate at room temperature, crystals of complexes **1**–**6** suitable for X-ray crystallography were obtained (Hope, 1994 ▸; Senge, 2000 ▸). The coordination compounds were characterized using X-ray crystallography, FT–IR spectroscopy and elemental analysis.

#### Analytical data for *L*   

M.p. 330 K. FT–IR (KBr, ν/cm^−1^, selected bands): 3445 (*w*, broad), 1246 (*s*), 3061 (*w*), 1615 (*s*), 792 (*s*), 849 (*m*).

#### Analytical data for 1   

Yield 52%. M.p. 505–507 K. FT–IR (KBr, ν/cm^−1^, selected bands): 3679 (*w*), 3447 (*w*), 1637 (*s*), 1525 (*m*), 1382 (*s*), 1025 (*m*), 800 (*m*), 750 (*m*). Analysis calculated for C_28_H_26_Cl_2_N_2_O_4_Zn (%): C 56.92, H 4.44, N 4.74; found: C 56.86, H 4.42, N 4.70.

#### Analytical data for 2   

Yield 70%. M.p. 520–522 K. FT–IR (KBr, ν/cm^−1^, selected bands): 3676 (*w*), 3447 (*w*), 1614 (*s*), 1542 (*m*), 1385 (*s*), 1019 (*m*), 805 (*m*), 754 (*m*). Analysis calculated for C_28_H_26_I_2_N_2_O_4_Zn (%): C 43.47, H 3.39, N 3.62; found: C 43.36, H 3.42, N 3.58.

#### Analytical data for 3   

Yield 54%. M.p. 543–545 K. FT–IR (KBr, ν/cm^−1^, selected bands): 3674 (*w*), 3445 (*m*), 1620 (*s*), 1530 (*m*), 1484 (*m*), 1383 (*s*), 1020 (*m*), 794 (*m*), 753 (*m*). Analysis calculated for C_28_H_26_Br_2_CdN_2_O_4_ (%): C 46.28, H 3.61, N 3.85; found: C 46.24, H 3.40, N 3.82.

#### Analytical data for 4   

Yield 46%. M.p. 461–463 K. FT–IR (KBr, ν/cm^−1^, selected bands): 3679 (*w*), 3447 (*m*), 1637 (*s*), 1520 (*m*), 1380 (*m*), 1025 (*m*), 796 (*m*), 741 (*m*). Analysis calculated for C_28_H_26_CdI_2_N_2_O_4_ (%): C 40.98, H 3.19, N 3.41; found: C 40.88, H 3.12, N 3.44.

#### Analytical data for 5   

Yield 65%. M.p. 438–440 K. FT–IR (KBr, ν/cm^−1^, selected bands): 3675 (*w*), 3435 (*m*), 1622 (*s*), 1534 (*m*), 1378 (*m*), 1028 (*m*), 794 (*m*), 750 (*m*). Analysis calculated for C_28_H_26_Cl_2_HgN_2_O_4_ (%): C 46.32, H 3.61, N 3.86; found: C 46.28, H 3.62, N 3.92.

#### Analytical data for 6   

Yield 70%. M.p. 383–385 K. FT–IR (KBr, ν/cm^−1^, selected bands): 3675 (*w*), 3446 (*m*), 1630 (*s*), 1523 (*m*), 1382 (*m*), 1018 (*m*), 796 (*m*), 738 (*w*). Analysis calculated for C_28_H_26_HgI_2_N_2_O_4_ (%): C 37.00, H 2.88, N 3.08; found: C 36.94, H 2.80, N 3.10.

### Refinement   

Crystal data, data collection and structure refinement details are summarized in Table 1 ▸. C- and N-bound H atoms were placed in their expected calculated positions and refined as riding, with N—H = 0.88 Å and C—H = 0.95–0.99 Å, and with *U*_iso_(H) = 1.5*U*_eq_(C) for methyl H atoms and 1.2*U*_eq_(N,C) otherwise. In the structure of **2**, the C and N atoms were restrained to have similar isotropic displacement parameters. Atoms N1*A*, N1*B* and C14*B* were restrained to have close to isotropic displacement parameters. The structure was solved as a rotational twin rotated from the first domain by 179.8° about the reciprocal axis 0.002 1.000 0.001 and the real axis 0.434 1.000 0.197. The twin law to convert *hkl* from the first to this domain (*SHELXL* TWIN matrix) was −0.999 0.004 −0.001, 0.866 0.998 0.395, 0.007 0.003 −0.999. The structure of **3** was solved as a rotational twin rotated from the first domain by 179.7° about the reciprocal axis −0.003 −0.997 1.000 and the real axis 0.311 1.000 −0.257. The twin law to convert *hkl* from the first to this domain (*SHELXL* TWIN matrix) was −1.001 0.001 −0.004, 0.498 0.590 −0.407, −0.487 −1.593 −0.589. The structure of **5** was solved as a rotational twin rotated from the first domain by 179.9° about the reciprocal axis −0.001 1.000 −0.999 and the real axis 0.345 1.000 −0.274. The twin law to convert *hkl* from the first to this domain (*SHELXL* TWIN matrix) was −1.000 −0.001 0.001, 0.541 0.570 −0.431, −0.543 −1.570 −0.570. The structure of **6** was solved as a rotational twin rotated from the first domain by 149.8° about the reciprocal axis 1.000 0.235 0.787 and the real axis 1.000 0.533 0.319. The twin law to convert *hkl* from the first to this domain (*SHELXL* TWIN matrix) was 0.534 0.949 0.308, 0.116 −0.693 0.359, 1.269 −0.145 −0.569.

## Results and discussion   

### Crystal structure analysis   

**Figure 1:**
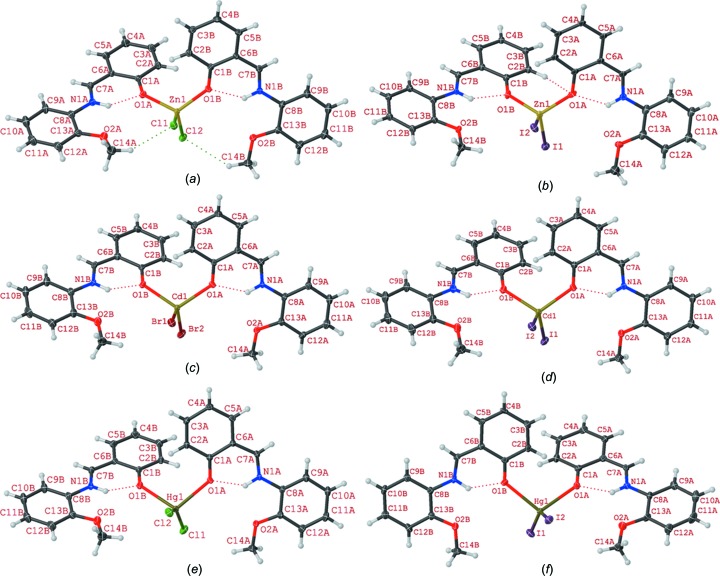
(*a*)–(*f*) The mol­ecular structures of compounds **1**–**6**, respectively, showing the atom labelling. Displacement ellipsoids are drawn at the 50% probability level.

X-ray crystallography revealed that compounds **1**–**6** are isostructural and crystallize in the triclinic space group *P* 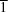 (Fig. 1 ▸ and Table 1 ▸). The asymmetric units of these structures contain two *L* ligands, two halide ions and a metal ion of group IIB. Crystal structure analysis reveals that in compounds **1**–**6**, the *M* ^II^ ion is in a distorted trigonal pyramidal geometry, with four-coordinate geometry indices, τ_4_ (Yang *et al.*, 2007 ▸), of 0.83, 0.85, 0.83, 0.84, 0.75, and 0.77, respectively. Selected bond lengths and angles are listed in Table 2 ▸ and are in agreement with the values reported for similar compounds (Shkol’nikova *et al.*, 1970 ▸; Gong *et al.*, 2014 ▸). The trigonal pyramidal geometry around *M* ^II^ is made up of two halide ions and two phenolate O atoms from two different *L* ligands. It should be noted that *N*-salicylideneanilines may exist in different tautomeric forms and the tautomeric isomerization reaction between the enol and keto forms is accompanied by intra- and inter­molecular proton transfer (Dürr & Bouas-Laurent, 2003 ▸; Cohen & Schmidt, 1962 ▸; Cohen *et al.*, 1964 ▸; Tsuchimoto *et al.*, 2016 ▸). The Schiff base ligand *L* shows a self-isomerization induced by an intra­molecular proton transfer from the hy­droxy O to the imine N atom through an O—H⋯N hydrogen bond (Hoshino *et al.*, 1988 ▸; Alarcón *et al.*, 1999 ▸). Thus, the ligand is a zwitterion with the negative and positive charges located at atoms O1*B* and N1*B*, respectively (Charland *et al.*, 1989 ▸; Redshaw *et al.*, 2013 ▸; Tsuchimoto *et al.*, 2016 ▸; Kargili *et al.*, 2014 ▸). This is supported by the geometry of the ligand and the unambiguous location of the H atom attached to atom N1*B*. The ligand almost keeps it coplanarity upon coordination; the dihedral angles between the planes of the two aromatic rings of ligand *L* lie in the range 4.10–10.68° for compounds **1**–**6** (see supporting information), which is a consequence of intra­molecular N—H⋯O hydrogen bonding. In this form, *L* can act as a monodentate ligand, where it is coordinated to the metal ion *via* the phenolate O atom. It should be noted that at basic pH, the *L* ligand may act as a tridentate ligand through the imine N, phenolate O and meth­oxy O atoms (Gong *et al.*, 2014 ▸; Song *et al.*, 2013 ▸; Reddy *et al.*, 2003*a* ▸).

**Figure 2:**
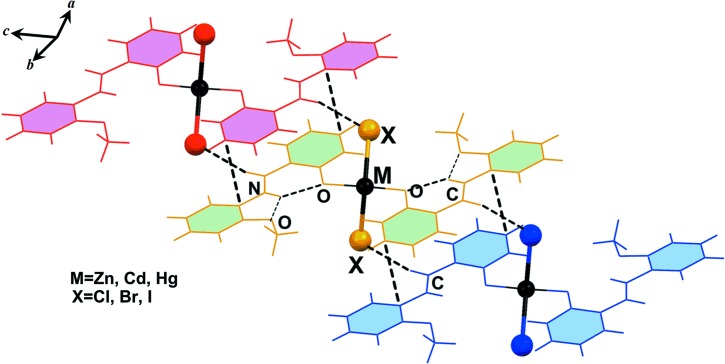
Representation of the self-assembly of compounds **1**–**6**, showing the association of discrete units through π–π stacking inter­actions in the crystallographic *a* direction and C—H⋯*X* (*X* = Cl, Br and I) hydrogen bonding in the *bc* plane.

**Figure 3:**
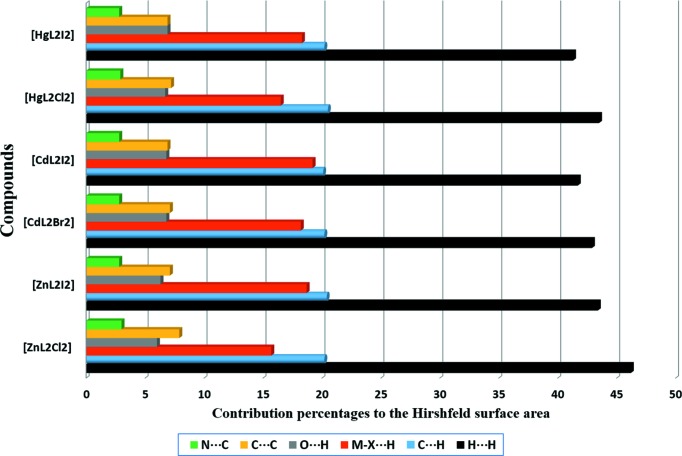
Relative contributions of the various noncovalent contacts to the Hirshfeld surface area in complexes **1**–**6**.

As shown in Fig. 2 ▸, the crystal packing of compounds **1**–**6** consists of mononuclear units which are connected in the crystallographic *a* direction through a combination of π–π stacking inter­actions involving the C=N group of the ligand and C—H⋯π inter­actions. These units are then linked to other units *via* C—H⋯*X* (*X* = Cl, Br and I) hydrogen-bonding inter­actions in the *bc* plane. The inter­molecular contacts involved in the crystal packing of compounds **1**–**6** can be qu­anti­fied *via* Hirshfeld surface analysis (Spackman & Jayatilaka, 2009 ▸; Mackenzie *et al.*, 2017 ▸). The analysis shows that in compounds **1**–**6**, the H⋯H inter­actions have the highest priority (the highest contribution to the Hirshfeld surface) and the C—H⋯π, *M*—*X*⋯H and π–π inter­actions have the next highest priorities, respectively. Also, it has been found that the probability of hydrogen-bonding *M*—*X*⋯H inter­actions involving metal-bound halogen increases for a given metal on going from a lighter to a heavier halogen atom. Selected contribution percentages are shown as a histogram in Fig. 3 ▸.

### Theoretical study   

Six *ML*_2_ *X*_2_ (*M* = Zn, Cd or Hg and *X* = Cl, Br or I) complexes have been synthesized and characterized by X-ray diffraction analysis (see Fig. 1 ▸). The ligand is monocoordinated to the metal centre and presents an extended π-system that comprises two phenyl rings and an imino group that connects both aromatic moieties.

**Figure 4:**
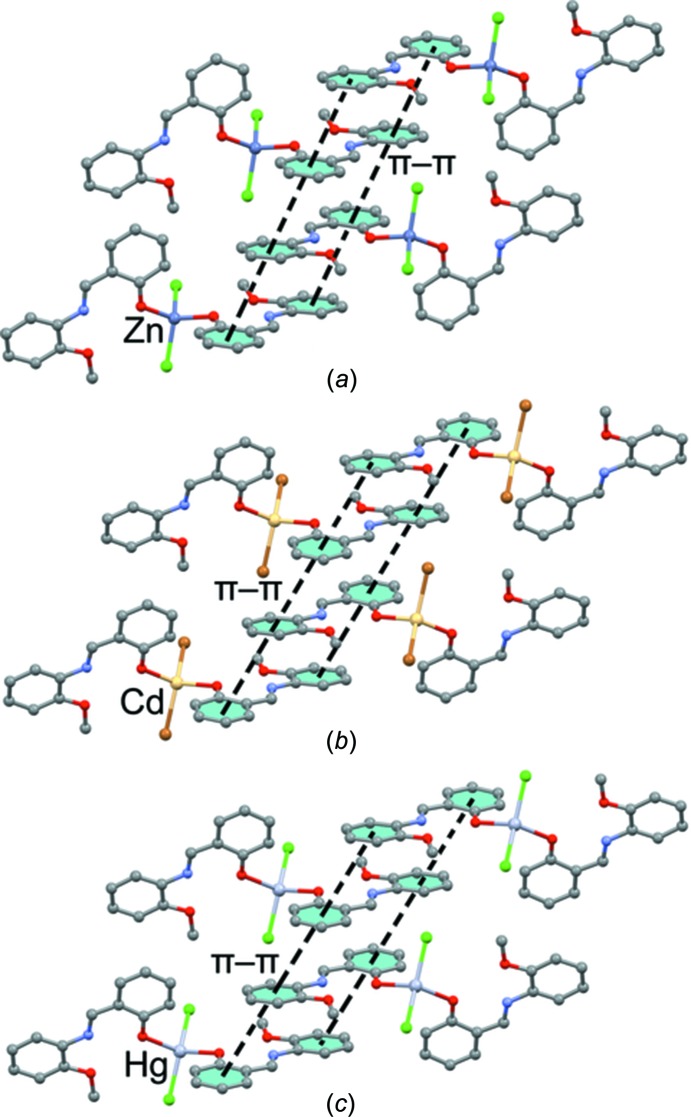
Partial view of the X-ray crystal structures in compounds (*a*) **1** (Zn), (*b*) **3** (Cd) and (*c*) **5** (Hg).

The solid-state architecture of all six structures is governed by the formation of π-stacking inter­actions between the aromatic ligands. In particular, each ligand forms infinite one-dimensional (1D) ladders in the crystal packing, as detailed for compounds **1**, **3** and **5** in Fig. 4 ▸ as representative systems.

**Figure 5:**
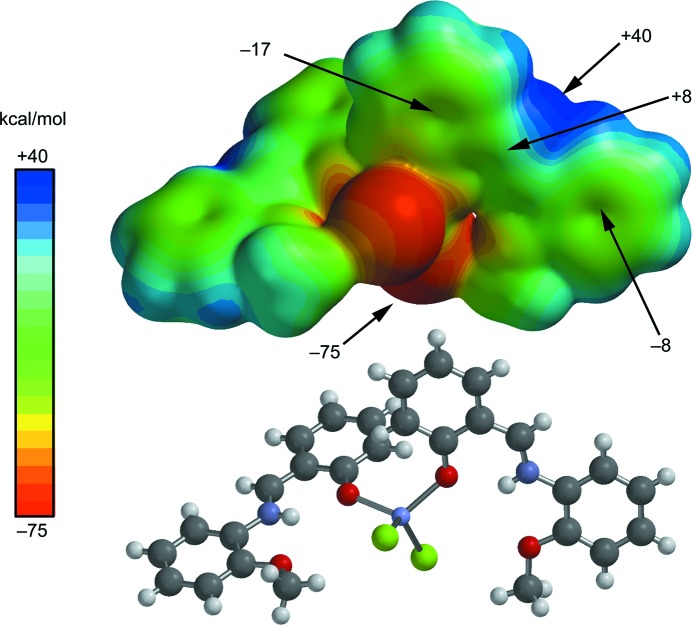
MEP surface of compound **1**. The MEP values at selected points are given in kcal mol^−1^.

We have focused the theoretical study on a comparison of the energetic features shown by the π-stacking and hydrogen-bonding inter­actions (depending on the type of metal) observed in the crystal packing of compounds **1**–**6** described above. In particular, we have analyzed the π–π and C—H⋯*X* noncovalent inter­actions that are crucial to understanding their solid-state architectures. First of all, in order to study the donor–acceptor ability of the *ML*_2_ *X*_2_ complexes, we have computed the mol­ecular electrostatic potential (MEP) surface of a model system (compound **1**), which is shown in Fig. 5 ▸. As expected, the most negative electrostatic potential corresponds to the region of the Cl ligands (−75 kcal mol^−1^). The MEP surface also reveals that the N—H group is totally inaccessible since it is involved in an intra­molecular hydrogen bond with the O atom of the ligand. Consequently, the most positive part is located in the region of the exocyclic C—H group at the mol­ecular plane, also influenced by the aromatic C—H groups (40 kcal mol^−1^). Therefore, hydrogen-bonding inter­actions between these groups (C—H⋯*X*) should be electrostatically favoured. Furthermore, perpendicular to the mol­ecular plane, we found that each aromatic ring presents negative MEP values (−17 and −8 kcal mol^−1^); therefore, face-to-face π–π stacking inter­actions are not electrostatically favoured (electrostatic repulsion). Remarkably, the electrostatic potential over the π-system of the linker (C=N) is positive, thus explaining the large displacement observed in the anti­parallel π-stacking inter­actions highlighted in Fig. 4 ▸ and further discussed below.

**Figure 6:**
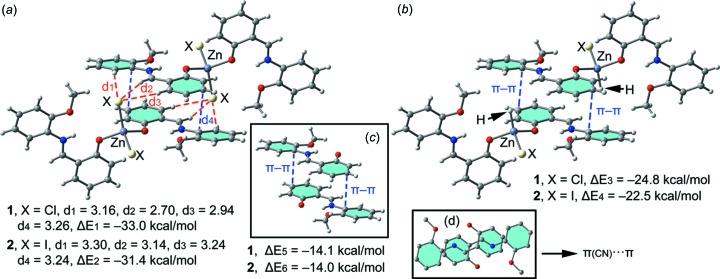
(*a*) Inter­action energies of the self-assembled π-stacked dimers observed in the solid state of compounds **1** and **2**. (*b*)/(*c*) Inter­action energies in several theoretical models of **1** and **2**. (*d*) On-top representation of the π-stacking inter­action. All distances are in Å.

In isostructural Zn compounds **1**–**3**, we have computed the inter­action energies of the self-assembled π-stacked dimers (shown in Fig. 6 ▸ *a*) that are responsible for the formation of the 1D ladders shown in Fig. 3 ▸. The self-assembled dimers are stabilized by a combination of hydrogen bonds and π–π stacking inter­actions involving the C=N group of the ligand. The dimerization energies in **1** and **2** (Δ*E*1 = −33.0 kcal mol^−1^ and Δ*E*2 = −31.4 kcal mol^−1^, respectively) are very large due to the contribution of both hydrogen-bonding (red dashed lines in Fig. 6 ▸) and π–π inter­actions (blue dashed lines in Fig. 6 ▸), where the former involves the most positive (C—H groups, see Fig. 5 ▸) and the most negative (belts of the halide ligands) potential regions of the metal compound. In an effort to calculate the contribution of the different forces that govern the formation of the self-assembled dimers, we have computed additional theoretical models where the halide ligands that establish the hydrogen bonds have been replaced by hydride ligands (see Fig. 6 ▸ *b*) and consequently the hydrogen-bonding inter­actions between the halide ligands and the C—H groups are not formed. As a result, the inter­action energies are reduced to Δ*E*3 = −24.8 kcal mol^−1^ and Δ*E*4 = −22.5 kcal mol^−1^ in **1** and **2**, respectively. Therefore, the contribution of both symmetrically equivalent hydrogen-bonding inter­actions can be roughly estimated by the difference (they are −8.2 and 8.9 kcal mol^−1^ for **1** and **2**, respectively) and it is similar in both compounds. Furthermore, we have used additional dimers where the ZnCl_2_ group in **1** or the ZnI_2_ group in **2** has been removed (see Fig. 6 ▸ *c*) in order to evaluate the influence of the metal coordination on the inter­action energy. The resulting inter­action energies are almost identical for both complexes (Δ*E*5 = −14.0 kcal mol^−1^ and Δ*E*6 = −14.1 kcal mol^−1^ for **1** and **2**, respectively) and reveal the strong influence of the metal coordination on the π–π stacking inter­action. This is likely due to the stronger dipole–dipole inter­action in the anti­parallel arrangement of the assembly. It is also worthy to mention that the π–π inter­action energy computed for these compounds is large compared to other π-stacking inter­actions (*i.e.* benzene dimer). This is due to the special arrangement of the two π-systems where the C=N bond is located over the aromatic ring (see the on-top representation in Fig. 6 ▸). This fact is in very good agreement with the MEP surface represented in Fig. 5 ▸ and explains the large inter­action energy since two electrostatically enhanced π(CN)⋯π inter­actions are established.

**Figure 7:**
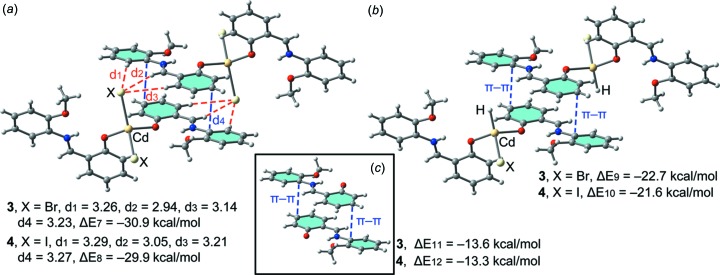
(*a*) Inter­action energies of the self-assembled π-stacked dimers observed in the solid state of compounds **3** and **4**. (*b*)/(*c*) Inter­action energies in several theoretical models of **3** and **4**. All distances are in Å.

In Cd compounds **3** and **4**, the π-stacking binding mode is very similar to that described before for **1** and **2**. As mentioned above, hydrogen-bonding and π–π inter­actions control the dimer formation (see Fig. 7 ▸ *a*). The computed inter­action energies of the self-assembled dimers are almost identical (Δ*E*7 = −30.9 kcal mol^−1^ and Δ*E*8 = −29.9 kcal mol^−1^ for **3** and **4**, respectively), indicating that the halide (Br or I) has a minimal influence on the binding energy. Compared to **1** and **2**, the inter­action energies are less favourable, thus revealing a larger influence of the Zn ion on the binding energy of the assembly compared to Cd. Also, in both compounds, we have computed theoretical models where the Br or I ligands have been replaced by H atoms and consequently the hydrogen bonds are not formed (see Fig. 7 ▸ *b*). As a result, the inter­action energies are reduced to Δ*E*9 = −22.7 kcal mol^−1^ and Δ*E*10 = −21.6 kcal mol^−1^ in **3** and **4**, respectively. Therefore, this contribution (both hydrogen bonds) can be roughly estimated by the difference (−8.2 and −8.3 kcal mol^−1^ for **3** and **4**, respectively). These values are very close to those found for compounds **1** and **2**, thus indicating that the contribution of the hydrogen bonds is not influenced by the type of transition metal (Zn or Cd). Furthermore, we have used an additional dimer, where the CdBr_2_ and CdI_2_ groups have been removed. The inter­action energies are further reduced to Δ*E*9 = −13.3 kcal mol^−1^ and Δ*E*6 = −13.6 kcal mol^−1^ for **3** and **4**, respectively, which is in agreement with the Zn complexes, revealing a strong influence of the metal coordination on the strength of the π-stacking inter­action.

**Figure 8:**
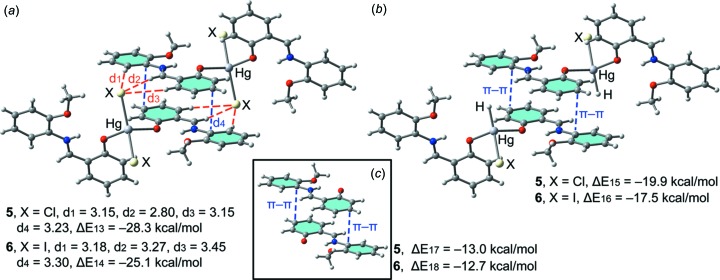
(*a*) Inter­action energies of the self-assembled π-stacked dimers observed in the solid state of compounds **5** and **6**. (*b*)/(*c*) Inter­action energies in several theoretical models of **5** and **6**. All distances are in Å.

For Hg compounds **5** and **6**, we have performed an equivalent study (see Fig. 8 ▸). The computed inter­action energies of the self-assembled dimers are almost identical (Δ*E*13 = −28.3 kcal mol^−1^ and Δ*E*14 = −25.1 kcal mol^−1^ for **5** and **6**, respectively), indicating that Hg has a smaller effect on the inter­action energy than Cd and Zn. Also, in both Hg compounds, we have computed theoretical models where the Cl^−^ or I^−^ ligands have been replaced by H^−^ ligands and consequently the hydrogen bonds are not formed (see Fig. 8 ▸ *b*). As a result, the inter­action energies are reduced to Δ*E*15 = −19.9 kcal mol^−1^ and Δ*E*16 = −17.5 kcal mol^−1^ in **5** and **6**, respectively. Therefore, this contribution (both hydrogen bonds) can be roughly estimated as −8.4 and −7.6 kcal mol^−1^ for **5** and **6**, respectively. These values are in agreement with those found for compounds **1**–**4**, thus confirming that the inter­action energy of the hydrogen bonds is not influenced by the type of transition metal (Zn/Cd/Hg). Furthermore, we have used an additional dimer, where the HgCl_2_ and HgI_2_ groups have been eliminated. Consequently, the inter­action energies are further reduced to Δ*E*17 = −13.0 kcal mol^−1^ and Δ*E*18 = −12.7 kcal mol^−1^ for **5** and **6**, respectively; which is in agreement to the rest of complexes commented on above and confirms the strong influence of the metal coordination on the strength of the π-stacking inter­action.

**Figure 9:**
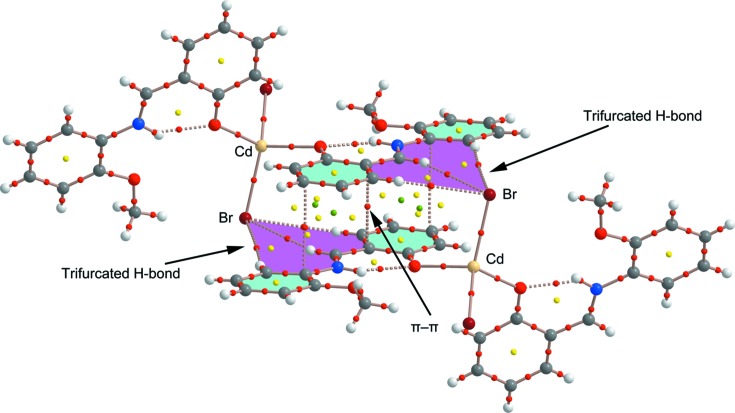
AIM analysis of the self-assembled dimers retrieved from the X-ray structure of compound **3**. Bond, ring and cage critical points are represented by red, yellow and green spheres, respectively. The bond paths connecting bond critical points are also represented by dashed lines.

In order to provide additional evidence for the existence of the C—H⋯*X* hydrogen-bond and π–π stacking inter­actions, we have analyzed the self-assembled π-stacked dimer of compound **3** (as an exemplifying model) using Bader’s theory of ‘atoms in mol­ecules’ (AIM) (Bader, 1991 ▸), which provides an unambiguous definition of chemical bonding. The AIM theory has been successfully used to characterize and understand a great variety of inter­actions, including those described herein. In Fig. 9 ▸ we show the AIM analysis of compound **3**. It can be observed that the π–π inter­action is characterized by the presence of three bond critical points that inter­connect three C atoms of each aromatic ligand. The inter­action is further characterized by several ring and cage critical points. Furthermore, the distribution of critical points reveals the existence of two symmetrically disposed sets of C—H⋯Br hydrogen-bonding inter­actions. Each one is characterized by a bond critical point and a bond path connecting one H atom of the C—H groups with the Br^−^ ligand, thus confirming the formation of the trifurcated hydrogen bonds. The value of the Laplacian at the bond critical points is positive, as is common in closed-shell inter­actions.

## Conclusion   

We herein reported the syntheses and structural characterization of six new metal complexes based on the 2-{[(2-meth­oxy­phen­yl)imino]­meth­yl}phenol ligand. All compounds exhibited an infinite 1D ladder in the solid state governed by the formation of hydrogen-bonding and π–π stacking inter­actions in the solid state. The crystal structure of these compounds was studied using geometrical and Hirshfeld surface analyses. They have also been studied using M06-2X/def2-TZVP calculations and Bader’s theory of ‘atoms in mol­ecules’. The energies associated with the inter­actions, including the contribution of the different forces, have been evaluated. In general, the π–π stacking inter­actions are stronger than those reported for conventional π–π complexes, that is attributed to the influence of the metal coordination, which is stronger for Zn than for either Cd or Hg. The results reported herein might be useful for understanding the solid-state architecture of metal-containing materials that contain *M* ^II^ *X*_2_ subunits and organic aromatic ligands.

## Supplementary Material

Crystal structure: contains datablock(s) Compound_1, Compound_2, Compound_3, Compound_4, Compound_5, Compound_6, global. DOI: 10.1107/S2053229618018314/sk3704sup1.cif

Structure factors: contains datablock(s) Compound_1. DOI: 10.1107/S2053229618018314/sk3704Compound_1sup2.hkl

Structure factors: contains datablock(s) Compound_2. DOI: 10.1107/S2053229618018314/sk3704Compound_2sup3.hkl

Structure factors: contains datablock(s) Compound_3. DOI: 10.1107/S2053229618018314/sk3704Compound_3sup4.hkl

Structure factors: contains datablock(s) Compound_4. DOI: 10.1107/S2053229618018314/sk3704Compound_4sup5.hkl

Structure factors: contains datablock(s) Compound_5. DOI: 10.1107/S2053229618018314/sk3704Compound_5sup6.hkl

Structure factors: contains datablock(s) Compound_6. DOI: 10.1107/S2053229618018314/sk3704Compound_6sup7.hkl

CCDC references: 1861558, 1861557, 1861556, 1861555, 1861554, 1861553

## Figures and Tables

**Table:** 

	**1**	**2**	**3**
Crystal data			
Chemical formula	[ZnCl_2_(C_28_H_26_N_2_O_4_)]	[ZnI_2_(C_28_H_26_N_2_O_4_)]	[CdBr_2_(C_28_H_26_N_2_O_4_)]
*M*_r_	590.78	773.68	726.73
Crystal system, space group	Triclinic, *P* 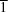	Triclinic, *P* 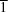	Triclinic, *P* 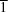
Temperature (K)	100	100	100
*a*, *b*, *c* (Å)	9.1926 (2), 10.6101 (2), 14.8057 (3)	9.2709 (19), 10.020 (2), 16.248 (4)	9.2772 (3), 10.0935 (3), 16.1021 (5)
α, β, γ (°)	94.188 (1), 97.716 (1), 114.409 (1)	98.56 (4), 100.50 (4), 110.09 (3)	97.699 (2), 100.586 (2), 111.149 (2)
*V* (Å^3^)	1289.80 (5)	1356.7 (6)	1348.92 (8)
*Z*	2	2	2
Radiation type	Mo *K*α	Mo *K*α	Mo *K*α
μ (mm^−1^)	1.20	3.22	3.81
Crystal size (mm)	0.26 × 0.12 × 0.11	0.14 × 0.07 × 0.03	0.27 × 0.13 × 0.10

Data collection			
Diffractometer	Bruker SMART APEXII area detector	Bruker APEXII area detector	Bruker APEXII area detector
Absorption correction	Multi-scan (*SADABS*; Bruker, 2016 ▸)	Multi-scan (*TWINABS*; Bruker, 2012 ▸)	Multi-scan (*TWINABS*; Bruker, 2012 ▸)
*T*_min_, *T*_max_	0.691, 0.747	0.612, 0.746	0.538, 0.745
No. of measured, independent and observed [*I* > 2σ(*I*)] reflections	93163, 11965, 9331	7281, 7281, 4608	10130, 10130, 8342
*R*_int_	0.053	0.116	0.020
(sin θ/λ)_max_ (Å^−1^)	0.822	0.606	0.634

Refinement			
*R*[*F* ^2^ > 2σ(*F* ^2^)], *wR*(*F* ^2^), *S*	0.035, 0.084, 1.02	0.067, 0.195, 0.99	0.040, 0.126, 1.03
No. of reflections	11965	7281	10130
No. of parameters	336	337	337
No. of restraints	0	174	0
H-atom treatment	H-atom parameters constrained	H-atom parameters constrained	H-atom parameters constrained
Δρ_max_, Δρ_min_ (e Å^−3^)	0.77, −0.71	2.19, −1.18	1.04, −0.70

**Table:** 

	**4**	**5**	**6**
Crystal data			
Chemical formula	[CdI_2_(C_28_H_26_N_2_O_4_)]	[HgCl_2_(C_28_H_26_N_2_O_4_)]	[HgI_2_(C_28_H_26_N_2_O_4_)]
*M*_r_	820.71	726.00	908.90
Crystal system, space group	Triclinic, *P* 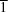	Triclinic, *P* 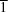	Triclinic, *P* 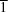
Temperature (K)	100	100	100
*a*, *b*, *c* (Å)	9.3200 (3), 10.0498 (3), 16.6239 (5)	9.2456 (4), 10.1510 (4), 15.8499 (6)	9.2783 (14), 10.0060 (15), 16.695 (3)
α, β, γ (°)	99.140 (1), 100.528 (1), 109.332 (1)	96.5447 (15), 99.7441 (15), 112.6735 (14)	98.777 (1), 100.296 (1), 109.396 (1)
*V* (Å^3^)	1403.58 (8)	1326.25 (9)	1400.4 (4)
*Z*	2	2	2
Radiation type	Mo *K*α	Mo *K*α	Mo *K*α
μ (mm^−1^)	3.01	6.04	7.74
Crystal size (mm)	0.40 × 0.26 × 0.14	0.17 × 0.14 × 0.08	0.38 × 0.19 × 0.13

Data collection			
Diffractometer	Bruker SMART APEXII area detector	Bruker APEXII area detector	Bruker APEXII area detector
Absorption correction	Numerical (*SADABS*; Bruker, 2016 ▸)	Multi-scan (*TWINABS*; Bruker, 2012 ▸)	Multi-scan (*TWINABS*; Bruker, 2012 ▸)
*T*_min_, *T*_max_	0.416, 0.667	0.630, 0.746	0.441, 0.746
No. of measured, independent and observed [*I* > 2σ(*I*)] reflections	110757, 13807, 10917	22779, 22779, 21098	11185, 11185, 10132
*R*_int_	0.055	0.012	0.071
(sin θ/λ)_max_ (Å^−1^)	0.838	0.668	0.650

Refinement			
*R*[*F* ^2^ > 2σ(*F* ^2^)], *wR*(*F* ^2^), *S*	0.033, 0.067, 1.03	0.022, 0.052, 1.03	0.032, 0.112, 1.08
No. of reflections	13807	22779	11185
No. of parameters	336	337	337
No. of restraints	0	0	0
H-atom treatment	H-atom parameters constrained	H-atom parameters constrained	H-atom parameters constrained
Δρ_max_, Δρ_min_ (e Å^−3^)	1.04, −1.33	1.29, −0.60	1.48, −1.78

Computer programs: *APEX3* (Bruker, 2016 ▸), *SAINT* (Bruker, 2015 ▸), *SHELXT* (Sheldrick, 2015*b* ▸), *SHELXL2014* (Sheldrick, 2015*a* ▸) and *OLEX2* (Dolomanov *et al.*, 2009 ▸).

**Table 2:** Selected bond lengths (Å) and angles (°) for compounds **1**–**6**

Compound		**1**	**2**	**3**	**4**	**5**	**6**
Bond lengths	*M*1—*X*1	2.240 (4)	2.588 (3)	2.537 (1)	2.720 (1)	2.371 (1)	2.658 (1)
	*M*1—*X*2	2.243 (4)	2.568 (3)	2.545 (1)	2.710 (1)	2.369 (1)	2.654 (1)
	*M*1—O2*A*	1.985 (1)	1.990 (10)	2.216 (4)	2.231 (2)	2.259 (2)	2.387 (5)
	*M*1—O2*B*	1.983 (1)	1.990 (10)	2.225 (4)	2.216 (2)	2.356 (2)	2.378 (5)
Bond angles	*X*1—*M*1—*X*2	124.8 (2)	123.7 (1)	130.0 (1)	130.1 (1)	148.2 (1)	145.1 (1)
	O1*A*—*M*1—*X*1	103.9 (3)	104.0 (4)	104.4 (1)	103.7 (1)	98.3 (1)	102.0 (1)
	O1*A*—*M*1—*X*2	104.0 (3)	103.6 (4)	103.6 (1)	104.8 (1)	100.4 (1)	98.7 (1)
	O1*B*—*M*1—*X*1	105.5 (3)	106.2 (4)	104.8 (1)	102.9 (1)	98.8 (1)	98.6 (1)
	O1*B*—*M*1—*X*2	102.8 (3)	104.2 (4)	101.9 (1)	103.5 (1)	100.7 (1)	102.3 (1)
	O1*A*—*M*1—O1*B*	116.8 (4)	115.8 (5)	112.0 (2)	111.6 (1)	105.6 (1)	105.8 (2)

## References

[bb1] Ahlrichs, R., Bär, M., Häser, M., Horn, H. & Kölmel, C. (1989). *Chem. Phys. Lett.* **162**, 165–169.

[bb2] Alarcón, S., Pagani, D., Bacigalupo, J. & Olivieri, A. (1999). *J. Mol. Struct.* **475**, 233–240.

[bb3] Azhdari Tehrani, A., Abedi, S. & Morsali, A. (2016). *Cryst. Growth Des.* **17**, 255–261.

[bb4] Bader, R. F. (1991). *Chem. Rev.* **91**, 893–928.

[bb5] Blake, A. J., Champness, N. R., Hubberstey, P., Li, W.-S., Withersby, M. A. & Schröder, M. (1999). *Coord. Chem. Rev.* **183**, 117–138.

[bb6] Boys, S. F. & Bernardi, F. D. (1970). *Mol. Phys.* **19**, 553–566.

[bb7] Braga, D. & Grepioni, F. (2000). *Acc. Chem. Res.* **33**, 601–608. 10.1021/ar990143u 10995197

[bb8] Braga, D., Grepioni, F. & Desiraju, G. R. (1998). *Chem. Rev.* **98**, 1375–1406. 10.1021/cr960091b 11848937

[bb9] Brammer, L. (2004). *Chem. Soc. Rev.* **33**, 476–489.

[bb10] Bruker (2012). *TWINABS*. Bruker AXS Inc., Madison, Wisconsin, USA.

[bb11] Bruker (2015). *SAINT*. Bruker AXS Inc., Madison, Wisconsin, USA.

[bb12] Bruker (2016). *APEX3* and *SADABS*. Bruker AXS Inc., Madison, Wisconsin, USA.

[bb13] Busschaert, N., Caltagirone, C., Van Rossom, W. & Gale, P. A. (2015). *Chem. Rev.* **115**, 8038–8155. 10.1021/acs.chemrev.5b00099 25996028

[bb14] Charland, J., Gabe, E., Khoo, L. & Smith, F. (1989). *Polyhedron*, **8**, 1897–1901.

[bb15] Cohen, M. & Schmidt, G. (1962). *J. Phys. Chem.* **66**, 2442–2446.

[bb16] Cohen, M., Schmidt, G. & Flavian, S. (1964). *J. Chem. Soc*. pp. 2041–2051.

[bb17] Custelcean, R., Bonnesen, P. V., Duncan, N. C., Zhang, X., Watson, L. A., Van Berkel, G., Parson, W. B. & Hay, B. P. (2012). *J. Am. Chem. Soc.* **134**, 8525–8534. 10.1021/ja300677w 22545671

[bb18] Đaković, M., Soldin, Ž., Kukovec, B.-M., Kodrin, I., Aakeröy, C. B., Baus, N. & Rinkovec, T. (2018). *IUCrJ*, **5**, 13–21. 10.1107/S2052252517015494 PMC5755573 29354267

[bb19] Desiraju, G. (1998). *Chem. Commun.* pp. 891–892.

[bb20] Desiraju, G. R. (2014). *Angew. Chem. Int. Ed.* **53**, 604–605.

[bb21] Dolomanov, O. V., Bourhis, L. J., Gildea, R. J., Howard, J. A. K. & Puschmann, H. (2009). *J. Appl. Cryst.* **42**, 339–341.

[bb22] Dürr, H. & Bouas-Laurent, H. (2003). In *Photochromism: Molecules and Systems*. Amsterdam: Elsevier.

[bb23] Gong, D., Wang, B., Jia, X. & Zhang, X. (2014). *Dalton Trans.* **43**, 4169–4178. 10.1039/c3dt52708e 24468706

[bb24] Hajiashrafi, T., Kharat, A. N., Love, J. A. & Patrick, B. O. (2013). *Polyhedron*, **60**, 30–38.

[bb25] Hajiashrafi, T., Ziarani, G. M., Kubicki, M., Fadaei, F. T. & Schenk, K. J. (2016). *Polyhedron*, **119**, 260–266.

[bb27] Holliday, B. J. & Mirkin, C. A. (2001). *Angew. Chem. Int. Ed.* **40**, 2022–2043. 11433436

[bb28] Hope, H. (1994). *Prog. Inorg. Chem.* **41**, 1–19.

[bb29] Hoshino, N., Inabe, T., Mitani, T. & Maruyama, Y. (1988). *Bull. Chem. Soc. Jpn*, **61**, 4207–4214.

[bb30] Janiak, C. (2000). *J. Chem. Soc. Dalton Trans.* pp. 3885–3896.

[bb26] Kargili, H., Alpaslan, G., Macit, M., Erdönmez, A. & Büyükgüngör, O. (2014). *Opt. Spectrosc.* **116**, 179–186.

[bb31] Keith, T. A. (2013). *AIMAll*. Version 13.05.06. TK Gristmill Software, Overland Park, KS, USA.

[bb32] Khavasi, H. R. & Azhdari Tehrani, A. (2013). *Inorg. Chem.* **52**, 2891–2905. 10.1021/ic3021113 23441758

[bb33] Khavasi, H. R. & Azizpoor Fard, M. (2010). *Cryst. Growth Des.* **10**, 1892–1896.

[bb34] Khavasi, H. R., Barforoush, M. M. & Fard, M. A. (2012). *CrystEngComm*, **14**, 7236–7244.

[bb35] Khavasi, H. R. & Mohammad Sadegh, B. M. (2010). *Inorg. Chem.* **49**, 5356–5358. 10.1021/ic100604k 20496920

[bb36] Khavasi, H. R., Norouzi, F. & Azhdari Tehrani, A. (2015). *Cryst. Growth Des.* **15**, 2579–2583.

[bb37] Khavasi, H. R. & Sadegh, B. M. M. (2014). *Dalton Trans.* **43**, 5564–5573. 10.1039/c3dt53220h 24549003

[bb38] Kielmann, M. & Senge, M. O. (2018). *Angew. Chem. Int. Ed.* **58**, 418–441. 10.1002/anie.201806281 PMC6391963 30067890

[bb39] Lehn, J.-M. (1995). In *Supramolecular Chemistry*. Weinheim: VCH.

[bb40] Li, B., Zang, S.-Q., Wang, L.-Y. & Mak, T. C. (2016). *Coord. Chem. Rev.* **308**, 1–21.

[bb41] Li, L. & Yuan, F. (2012). *Synth. React. Inorg. Met.-Org. Nano-Met. Chem.* **42**, 994–998.

[bb42] Mackenzie, C. F., Spackman, P. R., Jayatilaka, D. & Spackman, M. A. (2017). *IUCrJ*, **4**, 575–587. 10.1107/S205225251700848X PMC5600021 28932404

[bb43] Mahata, P., Prabu, M. & Natarajan, S. (2009). *Cryst. Growth Des.* **9**, 3683–3691.

[bb44] McKinlay, A. C., Morris, R. E., Horcajada, P., Férey, G., Gref, R., Couvreur, P. & Serre, C. (2010). *Angew. Chem. Int. Ed.* **49**, 6260–6266. 10.1002/anie.201000048 20652915

[bb45] Politzer, P., Murray, J. S. & Clark, T. (2010). *Phys. Chem. Chem. Phys.* **12**, 7748–7757. 10.1039/c004189k 20571692

[bb46] Reddy, P. A., Nethaji, M. & Chakravarty, A. R. (2003*a*). *Eur. J. Inorg. Chem.* pp. 2318–2324.

[bb47] Reddy, P. A., Nethaji, M. & Chakravarty, A. R. (2003*b*). *Inorg. Chem. Commun.* **6**, 698–701.

[bb48] Redshaw, C., Walton, M., Clowes, L., Hughes, D. L., Fuller, A. M., Chao, Y., Walton, A., Sumerin, V., Elo, P. & Soshnikov, I. (2013). *Chem. Eur. J.* **19**, 8884–8899. 10.1002/chem.201300453 23681561

[bb49] Reedijk, J. (2009). *Eur. J. Inorg. Chem.* pp. 1303–1312.

[bb50] Reedijk, J. (2013). *Chem. Soc. Rev.* **42**, 1776–1783. 10.1039/c2cs35239g 22903152

[bb51] Schottel, B. L., Chifotides, H. T., Shatruk, M., Chouai, A., Pérez, L. M., Bacsa, J. & Dunbar, K. R. (2006). *J. Am. Chem. Soc.* **128**, 5895–5912. 10.1021/ja0606273 16637658

[bb52] Semeniuc, R. F., Reamer, T. J. & Smith, M. D. (2010). *New J. Chem.* **34**, 439–452.

[bb53] Senge, M. O. (2000). *Z. Naturforsch. Teil B*, **55**, 336–344.

[bb54] Sheldrick, G. M. (2015*a*). *Acta Cryst.* C**71**, 3–8.

[bb55] Sheldrick, G. M. (2015*b*). *Acta Cryst.* A**71**, 3–8.

[bb56] Shkol’nikova, L., Obodovskaya, A. & Shugam, E. (1970). *J. Struct. Chem.* **11**, 47–53.

[bb57] Song, X., Wang, Z., Zhao, J. & Hor, T. A. (2013). *Chem. Commun.* **49**, 4992–4994. 10.1039/c3cc40320c 23532051

[bb58] Spackman, M. A. & Jayatilaka, D. (2009). *CrystEngComm*, **11**, 19–32.

[bb59] Steed, J. W. & Atwood, J. L. (2013). In *Supramolecular Chemistry*. London: John Wiley & Sons.

[bb60] Tsuchimoto, M., Yoshida, N., Sugimoto, A., Teramoto, N. & Nakajima, K. (2016). *J. Mol. Struct.* **1105**, 152–158.

[bb61] Wang, Z. J., Clary, K. N., Bergman, R. G., Raymond, K. N. & Toste, F. D. (2013). *Nat. Chem.* **5**, 100–103. 10.1038/nchem.1531 23344446

[bb62] Wiester, M. J., Ulmann, P. A. & Mirkin, C. A. (2011). *Angew. Chem. Int. Ed.* **50**, 114–137. 10.1002/anie.201000380 20922725

[bb63] Yang, L., Powell, D. R. & Houser, R. P. (2007). *Dalton Trans.* pp. 955–964. 10.1039/b617136b 17308676

[bb64] Zeng, F., Ni, J., Wang, Q., Ding, Y., Ng, S. W., Zhu, W. & Xie, Y. (2010). *Cryst. Growth Des.* **10**, 1611–1622.

